# Advancing hypoparathyroidism treatment: FDA approval of Palopegteriparatide as a promising orphan drug

**DOI:** 10.1097/MS9.0000000000004734

**Published:** 2026-01-20

**Authors:** Syeda Rabiah Shahid, Kamran Shahid, Sikandar Ehsan, Maliha Khalid, Aminath Waafira

**Affiliations:** a Department of Medicine, Shaikh Khalifa Bin Zayed Al-Nahyan Medical And Dental College,Lahore, Karachi, Pakistan; b Department of Medicine, Jinnah Sindh Medical University, Karachi, Pakistan; c Department of Medicine, The Maldives National University, Malé, Maldives

**Keywords:** calcium homeostasis, FDA approval, hypoparathyroidism, orphan drug, Palopegteriparatide, parathyroid hormone, Yorvipath

## Abstract

Hypoparathyroidism (HypoPT) is a rare endocrine disorder characterized by insufficient parathyroid hormone (PTH) levels, leading to hypocalcemia and hyperphosphatemia. Affecting approximately 70 000–90 000 individuals in the United States, HypoPT can arise from genetic mutations or, most commonly, as a result of surgical removal of the parathyroid glands. Traditional treatments involve calcium and vitamin D supplementation, which can pose long-term risks such as renal complications. On 9 August 2024, the U.S. Food and Drug Administration (FDA) approved Palopegteriparatide (Yorvipath, Ascendis, Denmark), a synthetic long-acting PTH analog, for subcutaneous use in adult patients with chronic HypoPT. Palopegteriparatide is designed as a pro-drug that undergoes auto-cleavage, offering sustained release and prolonged systemic exposure to recombinant PTH (1–34). Clinical trials have demonstrated its ability to maintain normocalcemia with minimal need for supplemental calcium or vitamin D. The treatment also showed a favorable safety profile, with mild transient side effects and no significant toxicity. Unlike standard-of-care therapies, Palopegteriparatide maintains balanced calcium-phosphate levels with less renal strain, making it a particularly promising option for patients with compromised kidney function. Although concerns remain regarding long-term safety, its FDA approval and orphan drug designation underscore its importance in addressing the unmet therapeutic needs of HypoPT. Continued investigation is warranted to optimize patient outcomes and further define its risk-benefit profile. This approval marks a critical milestone in endocrine therapeutics and offers renewed hope for patients struggling with the burdens of chronic HypoPT.

## Introduction

Hypoparathyroidism (HypoPT) is a rare endocrine disorder with a prevalence of approximately 37 per 100 000 individuals in the United States, characterized by low levels of parathyroid hormone (PTH), leading to hypocalcemia and hyperphosphatemia^[^1^]^. Its causes can vary from genetic mutations in the PTH, Glial Cells Missing B (GCMB), or calcium-sensing receptor genes – affecting PTH production – parathyroid gland development, or PTH secretion. Syndromic conditions like autoimmune polyglandular syndrome type 1 (APS1) can also lead to HypoPT, with other genetic mutations, such as GATA, GCMB, GNAS, and TBCE, further impairing parathyroid function^[^2,3^]^. However, the most common cause is the accidental removal of the parathyroid glands during neck surgeries like thyroid resection. Symptoms range from mild hair loss, tingling, and muscle cramps to severe complications like seizures, arrhythmias, and cataracts. Chronic HypoPT – persistent hypocalcemia for more than 6 months – can cause long-term problems including kidney dysfunction, cardiac abnormalities, bone disorders, and developmental delays in children. Its treatment primarily involves calcium and vitamin D supplementation, though this can strain kidney function over time. PTH replacement therapy offers another option, but managing the condition is a lifelong challenge, with patients facing both the ongoing need to maintain calcium balance and the financial burden of long-term care^[^4,5^]^.

HIGHLIGHTS

- Palopegteriparatide (Yorvipath^®^), a pegylated prodrug of synthetic parathyroid hormone (PTH; 1–34), mimics natural PTH action and has shown significant efficacy in maintaining normocalcemia, reducing urinary calcium loss, and improving quality of life without the need for high-dose calcium or vitamin D supplementation.
- Approved by the Food and Drug Administration in August 2024 for adult use, this once-weekly injectable offers a more physiologic, kidney-friendly alternative to conventional therapies, with a favorable safety profile observed in clinical trials, including the pivotal Phase 3 PaTHway study.
- Despite its orphan drug status and clinical promise, concerns remain regarding long-term safety (e.g., osteosarcoma risk), drug interactions, and cost-effectiveness; additional studies are urgently needed, particularly in pediatric and pregnant populations and in comparison with existing PTH analogs.

## Palopegteriparatide (Yorvipath^®^) – comprehensive drug profile

The US Food and Drug Administration (FDA) approved Palopegteriparatide (Yorvipath, Ascendis, Copenhagen, Denmark) as a treatment option for HypoPT with an orphan drug designation on 9 August 2024^[^6,7^]^. This injection is now approved for subcutaneous use in adults, marking a significant milestone in the management of hypoPT. This ultra-rare genetic disorder has long been a targeted treatment option, and the approval of Palopegteriparatide brings promising prospects for patients who have been suffering from the debilitating effects of this condition.

Palopegteriparatide is a synthetic inactive, pegylated PTH analog that undergoes controlled enzymatic cleavage. It closely mimics the biological effects of endogenous PTH by prolonged and stable stimulation of parathyroid hormone 1 receptor (PTH1R), predominantly found in the bones and kidneys. Through enhanced renal calcium reabsorption, reduced urinary calcium loss, increased phosphate excretion, and stimulation remodeling biased bone turnover, Palopegteriparatide helps regulate calcium levels in the body^[^8^]^.

**Figure 1.**
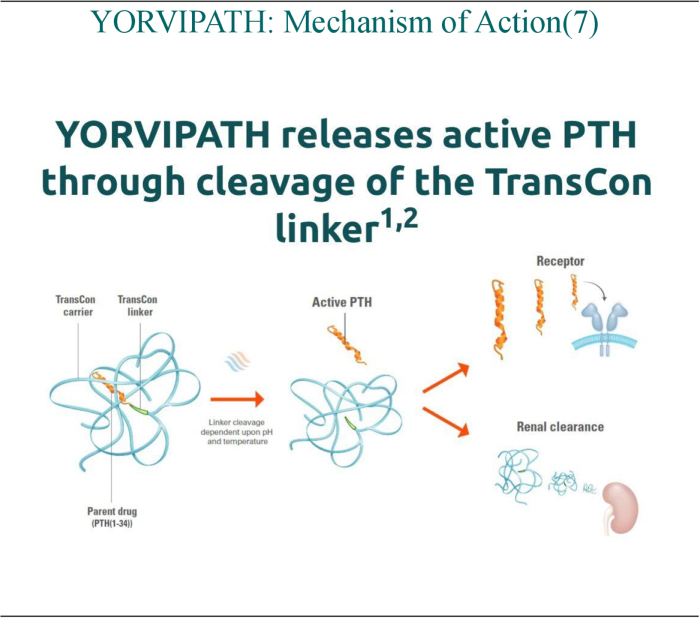
The mechanism of action of Palopegteriparatide (YORVIPATH).

Approved for the prophylaxis and treatment of chronic HypoPT, it is administered as a once-daily subcutaneous injection. Palopegteriparatide acts as a pegylated pro-drug, ensuring the sustained release of recombinant PTH (1–34) through auto-cleavage, which enhances its circulatory half-life^[^9^]^. Unlike conventional therapies, Palopegteriparatide does not rely solely on passive intestinal absorption of calcium, thus minimizing risk of hypercalciuria. Moreover, the stable, sustained release of PTH, lessens the pharmacological burden potentially leading to higher compliance and improved quality of life (QoL)^[^10–12^]^. Figure 1 shows the Mechanism of action of Palopegteriparatide (YORVIPATH).

## Clinical evidence

Several clinical trials, including the pivotal PaTHway trial (randomized double blind study), have demonstrated encouraging results of Palopegteriparatide as a potential replacement for conventional therapy with 79% of patients achieving the composite endpoint at 26 weeks compared to only 5% with placebo (*P* < 0.0001). This recombinant treatment shows notable efficacy in maintaining normal calcium levels (normocalcemia) in chronic HypoPT, often without requiring additional calcium or vitamin D supplementation^[^11,13^]^. Notable key observances included the significant reductions in 24-hour urinary excretion of calcium (60.7%), normal serum Calcium levels (92%), biochemical stability, and complete independence from active vitamin D supplementation at 52 week follow-up^[^11,13^]^. Normocalcemia was maintained in 86% patients while 95% of the patients attained complete independence from conventional therapy, along with significant improvements in renal function from baseline [mean difference of estimated Glomerular Filtration Rate (eGFR) = 9.3 (11.7) mL/min/1.73 m^2^, *P* < 0.0001].

The effects exhibited balanced calcium-phosphate levels with modest hypercalcemia at higher doses and minimal impact on bone turnover, evidenced by P1NP and CTX levels which remained within normal physiologic range^[^13,14^]^, making it a favorable option for patients with impaired kidney function^[^15,16^]^. Moreover, both Hypoparathyroid Patient Experience Scale and SF-36 scores, evaluating QoL in terms of fatigue, cognition, and physical functioning, were significantly improved in the treatment group (*P* < 0.05) as opposed to placebo^[^13^]^. These results are in contrast to standard care of treatment, which is known to increase the risk of renal damage^[^11^]^. Additionally, effective resolution of efficacy and safety concerns in prior trials, including the REPLACE trial, positions Palopegteriparatide as a landmark therapeutic breakthrough for patients with chronic, debilitating HypoPT^[^17^]^.

A safety profile with transient adverse effects and no severe toxicity was documented. Mild injection-site reactions, headache, and nausea were observed. Although animal studies associated the treatment with an increased risk of osteosarcoma, no such risks were observed at both short- and long-term follow-ups. None of the participants enrolled discontinued treatment owing to any treatment related adverse events^[^13,15^]^.

Table 1 shows the comparison of different PTH therapies.

**Table 1:** Comparison of different PTH therapies

Features	rhPTH (1–84) (Natpara)^[^11^]^	rhPTH (1–34) (Teriparatide)^[^13^]^	Palopegteriparatide (YORVIPATH)^[^8^]^
Status	FDA approved	Used off-label	FDA approved (2024)
Dosing frequency	Daily	Daily	Weekly
Mechanism	Full-length PTH	Fragment (1–34)	Pegylated prodrug (PTH 1–34)
Half-life	~3 hours	~1 hour	Sustained release over 7 days
Dose burden	Moderate	Moderate	Notably reduced
Urinary Ca^+2^ excretion	↓ (variable)	↓ (variable)	↓ Significantly
Supplemental vitamin D/calcium	Required (adjunct)	Required	Often unnecessary

## Limitations and future directions

Although the recent FDA approval of Palopegteriparatide has marked a significant achievement in the treatment of chronic HypoPT, there are impending concerns about long-term safety data, especially regarding skeletal consequences. Furthermore, interaction with other drugs^[^16^]^, borderless impact assessments, and the lack of data analyzing cost-effectiveness limit real-world utilization. Especially striking is that the pediatric and pregnant populations are not covered by the prescription at the moment, and there have not yet been any head-to-head trials of Palopetriperatide against rhPTH (1–84) either^[^18^]^. Trials establishing a safe and efficacious trajectory in pediatric populations are urgently necessary owing to particular vulnerability of this population to chronic hypocalcemia. Despite a value contribution assessment, long-term cost-effective and accessibility analyses are still warranted to ensure value for expense as an orphan drug^[^7^]^.

Regardless of these shortcomings, the FDA orphan drug designation sheds light on the clinical usefulness of the approved Palopegteriparatide^[^3^]^. This designation has brought with it developmental bonuses like tax credits, waived user fees, and even 7 years of guaranteed market exclusivity after the drug’s approval^[^3^]^. While these regulatory advances and its clinical outlook is promising, further research, particularly post distribution surveillance analyses, is essential to fully elucidate the long-term benefits and risks of Palopegteriparatide in optimizing treatment outcomes, health economics, and avoiding unnecessary complications^[^4,5^]^.

## Conclusion

In conclusion, the recent approval of Palopegteriparatide represents an important medical advancement in treating HypoPT. By targeting the root hormonal deficiency rather than relying on non-physiological supplementation, this once-weekly PTH analog offers the potential for biochemical stability, improves patient adherence, and reduces renal morbidity. Thus, this revolutionary drug, acting almost exactly like the missing PTH in the body, provides an optimistic outlook for patients suffering from this debilitating disease. Not only does this drug decrease the risk of renal impairment associated with conventional therapy of calcium and vitamin D, but it also ensures continuous systemic exposure to active PTH. Despite its potential side effects, the overall promising outcomes from its clinical trials highlight its effectiveness and potential in alleviating the demands of lifelong disease management; thus, improving the QoL for affected individuals. Furthermore, the therapeutic scope of Palopegteriparatide may extend beyond chronic HypoPT. Future research, especially the need for pediatric trials, is urgently warranted, due to the effects – developmental and neurocognitive – of poorly controlled hypocalcemia in children. Exploring its potential use in transient or post-surgical HypoPT, refractory cases of hypocalcemia in chronic kidney disease (CKD), or even as adjunctive therapy in rare disorders of calcium-phosphate metabolism could further be supported via extensive research and modifications in safety profile. Our work is in line with the TITAN Guidelines on the need for transparency in AI use in healthcare^[^19^]^.

## Data Availability

No data were generated for this manuscript.

## Research registration unique identifying number (UIN)

1. Name of the registry: N/A. 2. Unique Identifying number or registration ID: N/A. 3. Hyperlink to your specific registration (must be publicly accessible and will be checked): N/A.
